# Supplementary material for: Surface Bacterioplankton Community Structure Crossing the Antarctic Circumpolar Current Fronts
Source: Microorganisms. 2023 Mar 9;11(3):702. doi: 10.3390/microorganisms11030702 (PMC10054113; doi:10.3390/microorganisms11030702)
Supplement: Supplementary file 1 [file microorganisms-11-00702-s001.zip › microorganisms-2222266-supplementary/Supplementary figures and tables/Table S2.pdf]

Station	Phylum	Class	Order	Family	Genus	ASV Tot	Reads Tot
5	18	21	48	67	91	302	10873
6	17	20	49	68	93	310	12221
7	14	16	41	62	87	303	12561
10	14	17	42	63	92	314	9061
12	17	21	48	69	92	320	20838
15	9	11	28	45	62	182	9249
16	7	9	26	42	68	164	10496
17	9	11	31	52	83	207	7133
18	9	11	31	47	75	189	7399
19	10	13	33	55	87	224	12831
20	15	17	38	55	81	209	10399
23	5	7	19	31	53	116	2809
24	7	9	24	38	58	140	5036
