# Supplementary material for: Surface Bacterioplankton Community Structure Crossing the Antarctic Circumpolar Current Fronts
Source: Microorganisms. 2023 Mar 9;11(3):702. doi: 10.3390/microorganisms11030702 (PMC10054113; doi:10.3390/microorganisms11030702)
Supplement: Supplementary file 1 [file microorganisms-11-00702-s001.zip › microorganisms-2222266-supplementary/Supplementary figures and tables/Table S3.pdf]

Station	Observed	Shannon	Chao1	ASV	Covergae
**10**	314	4.33	339.57	314	0.92
**12**	320	4.31	334.54	320	0.96
**15**	182	3.60	200.45	182	0.91
**16**	164	3.27	193.33	164	0.85
**17**	207	3.78	239.50	207	0.86
**18**	189	3.45	228.14	189	0.83
**19**	224	3.60	243.56	224	0.92
**20**	209	3.53	245.12	209	0.85
**23**	116	3.47	133.11	116	0.87
**24**	140	3.51	158.40	140	0.88
**5**	302	4.44	323.00	302	0.93
**6**	310	4.27	328.50	310	0.94
**7**	303	4.36	324.58	303	0.93
