# Supplementary figures and images for: Surface Bacterioplankton Community Structure Crossing the Antarctic Circumpolar Current Fronts
Source: Microorganisms. 2023 Mar 9;11(3):702. doi: 10.3390/microorganisms11030702 (PMC10054113; doi:10.3390/microorganisms11030702)

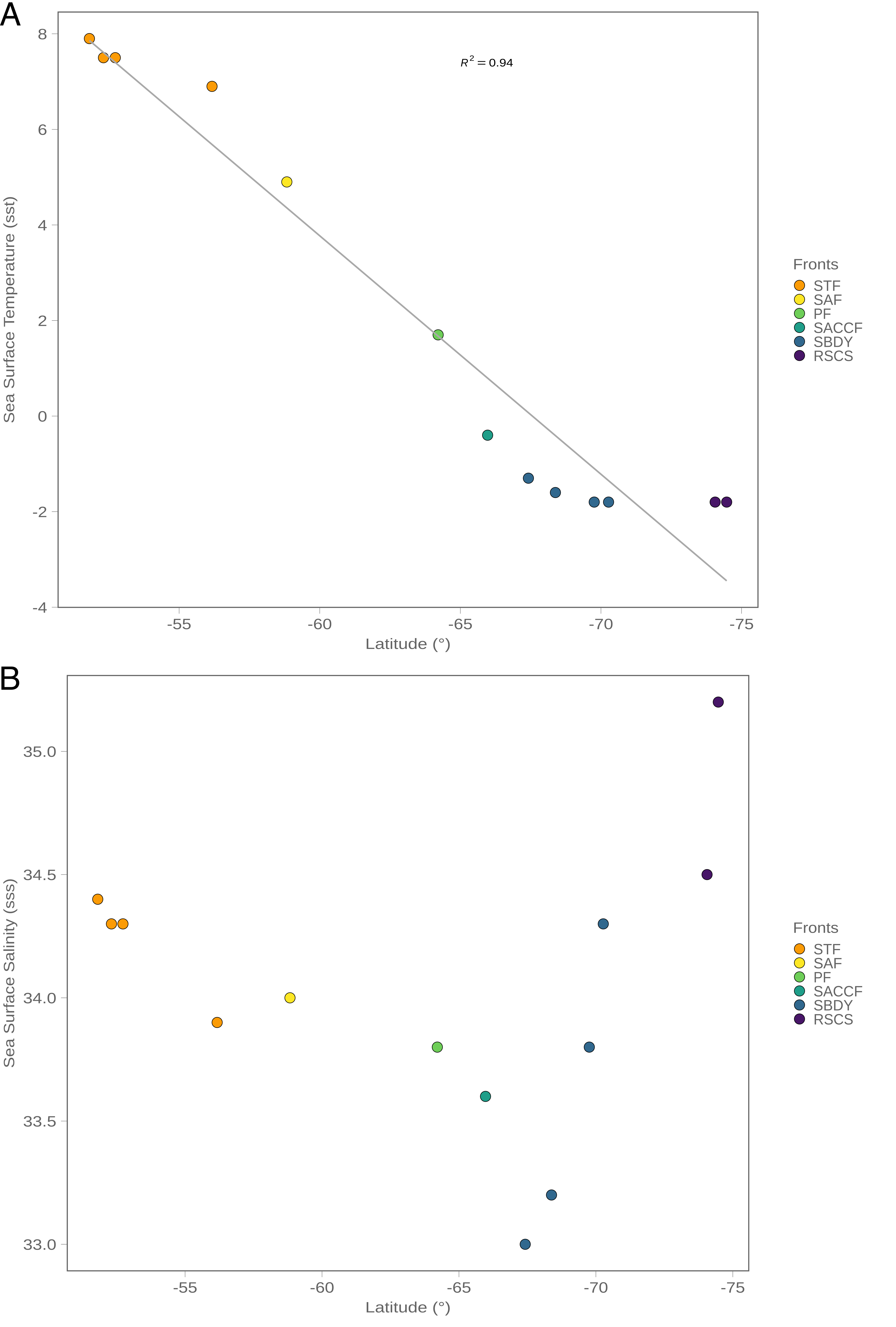

Supplement: Supplementary file 1 [file microorganisms-11-00702-s001.zip › microorganisms-2222266-supplementary/Supplementary figures and tables/Figure S1.png]

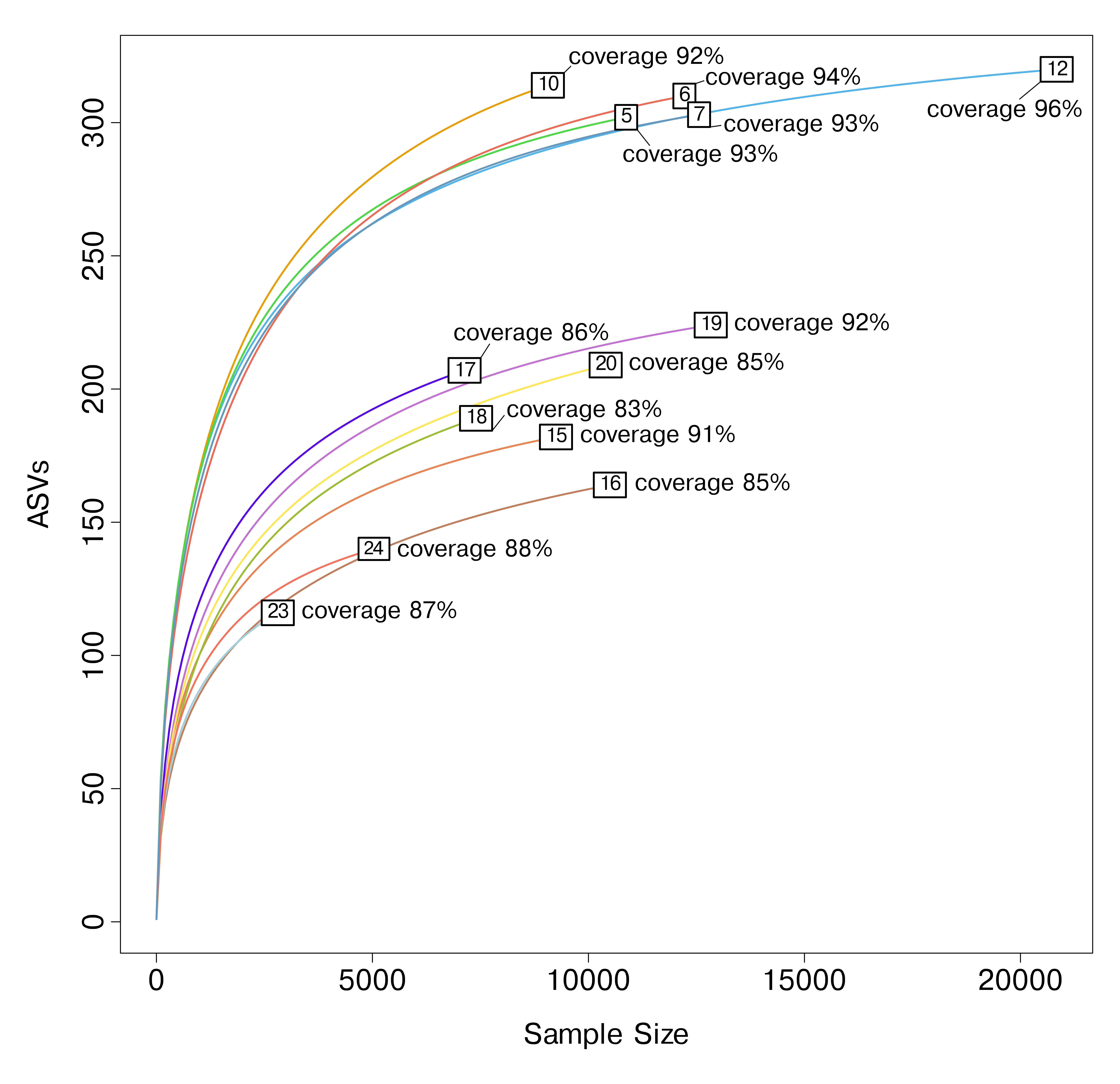

Supplement: Supplementary file 1 [file microorganisms-11-00702-s001.zip › microorganisms-2222266-supplementary/Supplementary figures and tables/Figure S2.png]

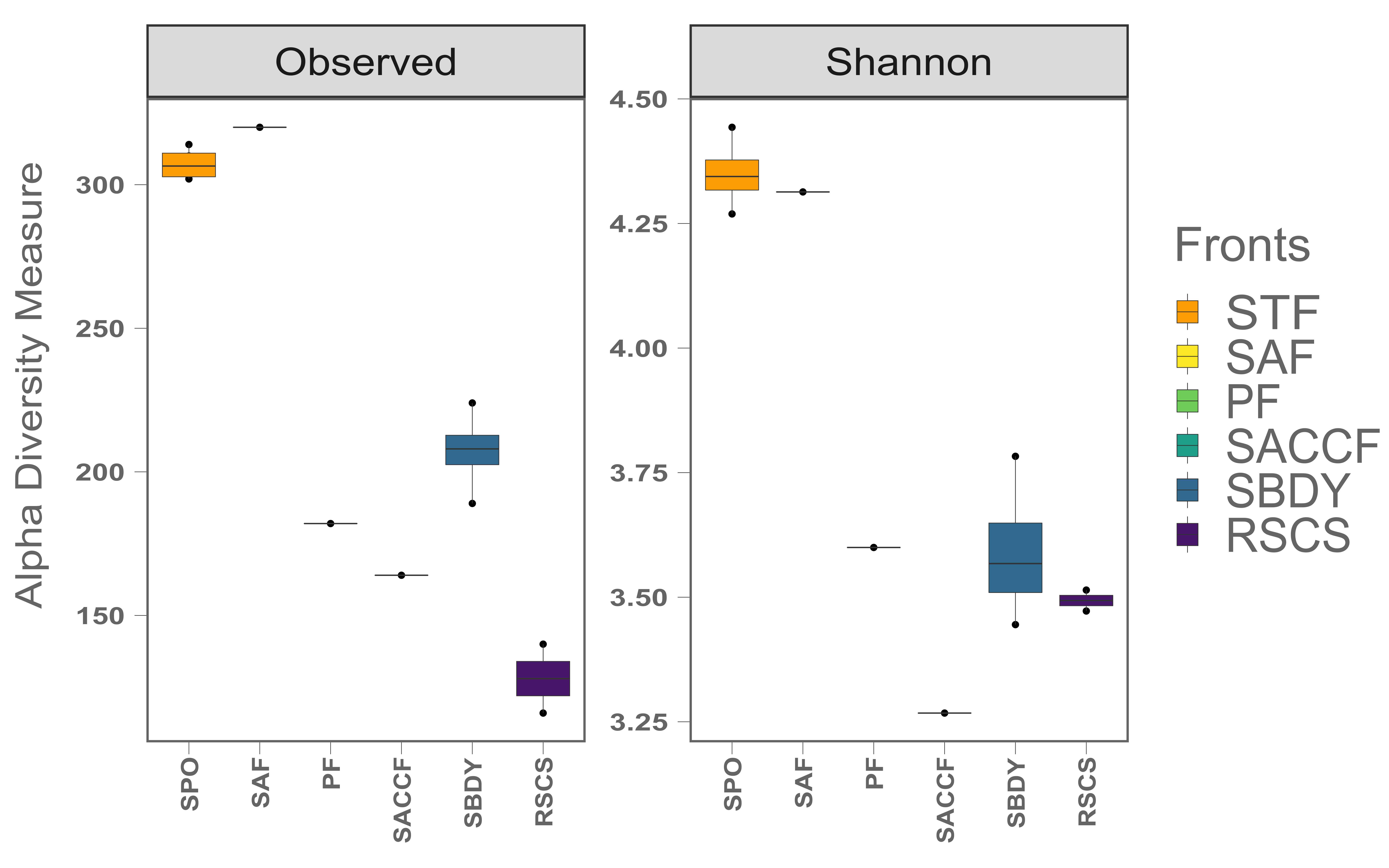

Supplement: Supplementary file 1 [file microorganisms-11-00702-s001.zip › microorganisms-2222266-supplementary/Supplementary figures and tables/Figure S3.png]

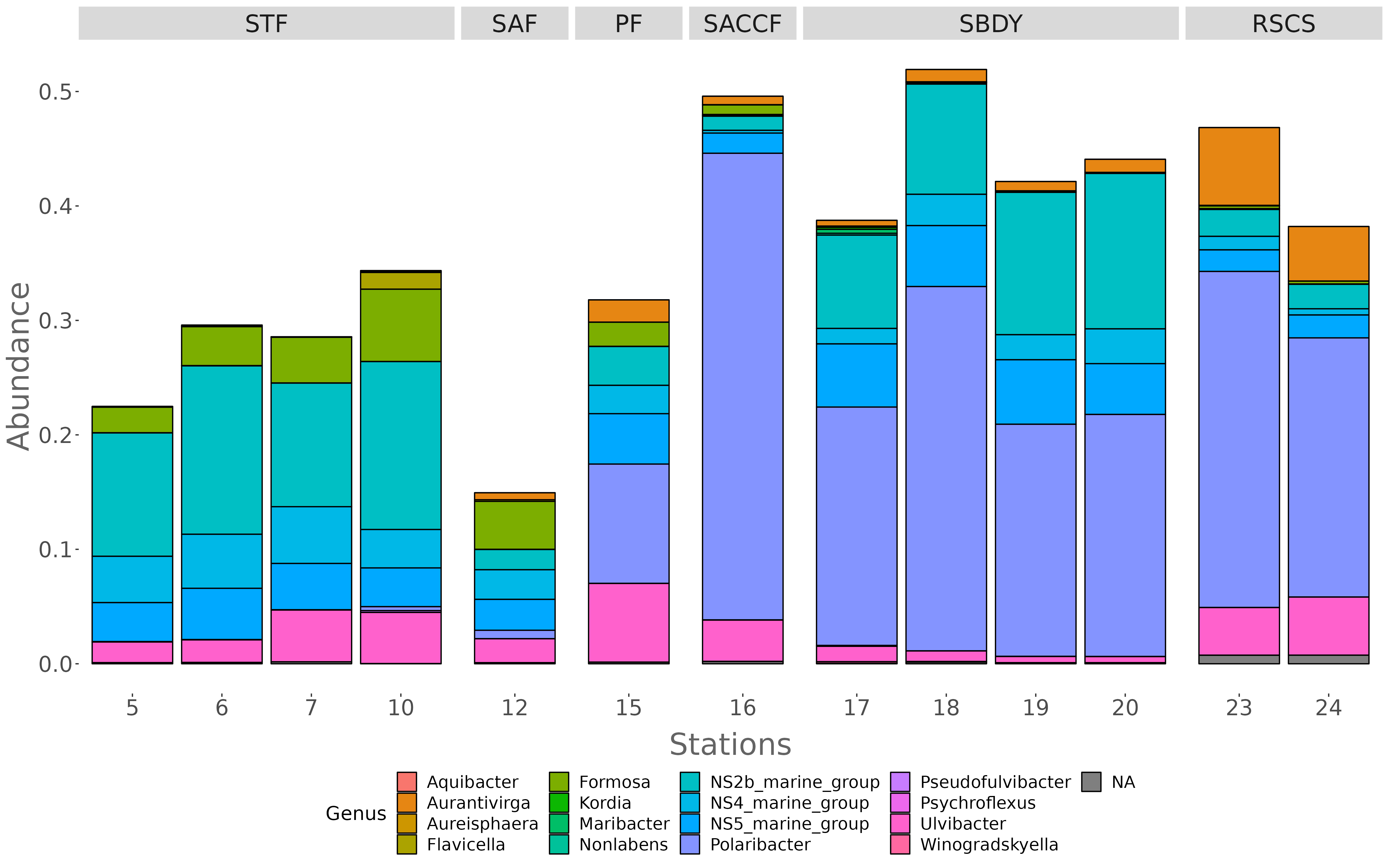

Supplement: Supplementary file 1 [file microorganisms-11-00702-s001.zip › microorganisms-2222266-supplementary/Supplementary figures and tables/Figure S4.png]
