# Supplementary material for: Surface Bacterioplankton Community Structure Crossing the Antarctic Circumpolar Current Fronts
Source: Microorganisms. 2023 Mar 9;11(3):702. doi: 10.3390/microorganisms11030702 (PMC10054113; doi:10.3390/microorganisms11030702)
Supplement: Supplementary file 1 [file Table S1.pdf › microorganisms-2222266-supplementary/Supplementary figures and tables/Table S1.pdf]

	Latitude	Longitude	SST	SSS	SPP	Prt_tot	Cho_tot	PN	POC	C_Dom	Chla	Chla_micro	Chla_nano	Chla_pico
Latitude	1	-0.31	0.97***	0	-0.29	-0.25	-0.46	-0.63*	-0.5	-0.68*	-0.51	-0.35	-0.31	0.42
Longitude	-0.31	1	-0.45	-0.85***	-0.61*	-0.35	0.71*	-0.18	-0.19	0.17	-0.14	0.88**	0.39	-0.8**
SST	0.97***	-0.45	1	0.19	-0.13	-0.16	-0.55	-0.55	-0.43	-0.62*	-0.41	-0.47	-0.29	0.49
SSS	0	-0.85***	0.19	1	0.66*	0.43	-0.42	0.3	0.3	0.11	0.27	-0.79**	-0.33	0.70**
SPP	-0.29	-0.61	-0.13	0.66*	1	0.83**	-0.06	0.78*	0.73*	0.53	0.62*	-0.47	-0.26	0.46
Prt_tot	-0.25	-0.35	-0.16	0.43	0.83***	1	0.23	0.86***	0.94**	0.72**	0.67*	-0.24	-0.11	0.22
Cho_tot	-0.46	0.71***	-0.55	-0.42	-0.06	0.23	1	0.27	0.31	0.67*	0.13	0.69	0.05	-0.46
PN	-0.63*	-0.18	-0.55	0.3	0.78*	0.86***	0.27	1	0.96***	0.81***	0.86***	-0.09	0.1	-0.01
POC	-0.5	-0.19	-0.43	0.3	0.73*	0.94***	0.31	0.96***	1	0.80***	0.77**	-0.09	0.05	0.02
C_Dom	-0.68*	0.17	-0.62*	0.11	0.53	0.72**	0.67*	0.81*	0.80*	1	0.66*	0.33	0.15	-0.3
Chla	-0.51	-0.14	-0.41	0.27	0.61*	0.67	0.13	0.86*	0.77*	0.66	1	-0.12	0.3	-0.12
Chla_micro	-0.35	0.88***	-0.47	-0.79**	-0.47	-0.24	0.69**	-0.09	-0.09	0.33	-0.12	1	0.23	-0.77**
Chla_nano	-0.31	0.39	-0.29	-0.33	-0.26	-0.11	0.05	0.1	0.05	0.15	0.3	0.23	1	-0.80**
Chla_pico	0.42	-0.80**	0.49	0.70**	0.46	0.22	-0.46	-0.01	0.02	-0.3	-0.12	-0.77**	-0.8	1

*. p-value < 0.05; **. p-value < 0.01; ***. p-value < 0.001
